# Long non-coding RNA NBR2 suppresses the progress of colorectal cancer in vitro and in vivo by regulating the polarization of TAM

**DOI:** 10.1080/21655979.2021.1958558

**Published:** 2021-09-10

**Authors:** Fuji Lai, Huiqin Zhang, Binbin Xu, Yangyang Xie, Hua Yu

**Affiliations:** a Department of Anus and Intestine Surgery, HuaMei Hospital, University of Chinese Academy of Sciences. No. 41 Xibei Street, Haishu District, Ningbo City, Zhejiang Province, China; b Ningbo Institute of Life and Health Industry, University of Chinese Academy of Sciences, No. 159 Beijiao Street, Jiangbei District, Ningbo City, Zhejiang Province, China; c Key Laboratory of Diagnosis and Treatment of Digestive System Tumors of Zhejiang Province, No. 41 Xibei Street, Haishu District, Ningbo City, Zhejiang Province, China; d Zhejiang Key Laboratory of Pathophysiology, Ningbo University. No. 818 Fenghua Street, Jiangbei District, Ningbo City Zhejiang Province, China

**Keywords:** LncRNA NBR 2, colorectal cancer, tumor associated macrophage, polarization

## Abstract

Colorectal cancer (CRC) threatens the health of patients with high mortality, which lacks sensitive biomarkers for diagnosis to improve total survival. The lncRNA NBR2 is reported to be downregulated in CRC and suppresses the proliferation of CRC cells. However, the underlying mechanisms remain unclear. The present study aimed to explore the regulatory function of the lncRNA NBR2 on tumor-associated macrophage (TAM) polarization and its consequent anti-tumor effect. Two CRC cell lines were used in this study. We found that the lncRNA NBR2, TNF-α, and HLA-DR were downregulated, and Arg-1, CD163, CD206, and IL-4 were upregulated in CRC tumors. M1 polarization was activated and M2 polarization was suppressed in NBR2-overexpressed macrophages, accompanied by increased production of inflammatory factors, decreased proliferation, and inhibited migration ability in the co-culture system of HCT-116 cells (SW480 cells) and NBR2-overexpressed macrophages. The promoted proliferation and migration were observed in the co-culture system of HCT-116 cells (SW480 cells) and NBR2-knockdown macrophages. The tumor growth of both HCT-116 cells and SW480 cells in the xenograft model was suppressed by co-planting NBR2-overexpressed macrophages and was facilitated by the co-planting of NBR2-knockdown macrophages. The release of inflammatory factors was induced, M1 polarization was facilitated, and M2 polarization was suppressed in tumor tissues in the NBR2-overexpressed group, which were all reversed in the NBR2-knockdown group. Therefore, the lncRNA NBR2 suppressed the progression of colorectal cancer *in vitro* and *in vivo* by regulating TAM polarization.

## Graphical abstract

**Figure:**
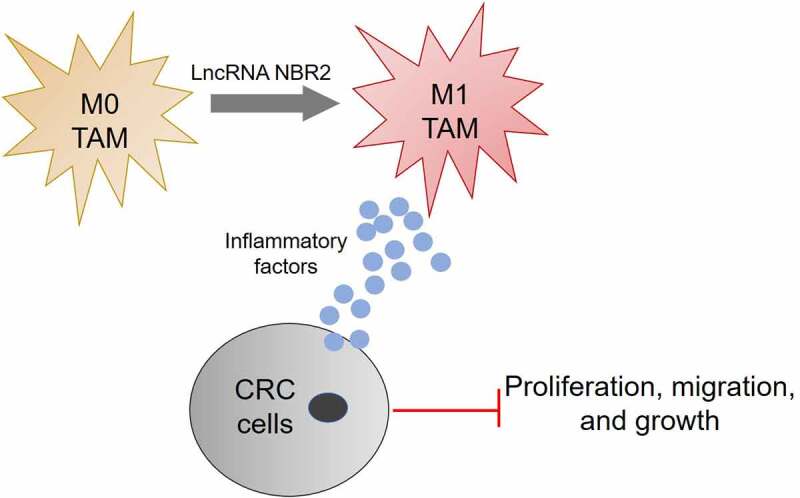

## Introduction

Colorectal cancer (CRC) is regarded as the second most common cause of cancer-related death, the mortality of which exceeds 600,000 every year globally [1]. Although the overall survival rate at the early stage has been promoted by improved strategies such as surgery, chemotherapy, and radiotherapy, immediate metastasis or relapses post-treatments were observed in approximately 40%–50% of diagnosed CRC patients [2]. Regular treatments are not suitable for most CRC patients with distant metastasis, which has a reported 5-year survival rate of less than 10% [3,4]. Therefore, exploring a more accurate strategy for the diagnosis of early-stage CRC is very important to reduce the morbidity of advanced CRC and prolong the overall survival rate of CRC patients.

The tumor microenvironment has been reported to be important for the process and development of malignant tumors. Tumor-associated macrophages (TAMs) are responsible for regulating the tumor microenvironment, which maintains the proliferation and metastasis tumor cells [5]. Activated TAMs can be differentiated into two types of macrophages. M1 macrophages are mainly produced by inducing the interferon gamma (IFN-γ) and lipopolysaccharides (LPS), which have been reported to exert important anti-tumor properties [6]. On the other hand, M2 macrophages are mainly differentiated from TAM by stimulating interleukin (IL)-4, IL-13, prostaglandin E_2_ (PGE2), or transforming growth factor (TGF)-β, which is reported to promote the proliferation and metastasis of tumor cells [7]. The activation of M2 polarization has also been observed in CRC processes and development [8].

Long noncoding RNA (lncRNA) is a type of noncoding RNA with a length of 200 nucleotides [9], which cannot code for proteins because of the lack of a complete reading frame [10]. Protein expression is influenced by lncRNA mainly at the transcriptional level, apparent modification level, and post-transcriptional level [11–13]. In addition, lncRNAs play an important role in the regulation of the tumor microenvironment, which has been proven by multiple studies [14,15]. It has been reported that lncRNAs are also involved in the regulation of macrophage polarization. Chen reported that lnc-M2 controlled M2 macrophage differentiation via the PKA/CREB pathway [16]. LncRNA GAS5 was found to promote M1 macrophage polarization via the miR-455-5p/SOCS3 pathway in childhood pneumonia [17]. Ye also reported that lncRNA cox-2 prevented immune evasion and metastasis of hepatocellular carcinoma by altering M1/M2 macrophage polarization [18]. The newly-discovered the lncRNA NBR2 has been reported to be involved in the process and development of multiple types of malignant tumors such as osteosarcoma [19], non-small-cell lung cancer [20] and thyroid cancer [21]. Based on data from public gene expression databases such as GEPIA, NBR2 is downregulated in colon cancer (https://gepia.cancer-pku.cn/detail.php?gene=NBR2). A previous study indicated that the lncRNA NBR2 is an important mediator involved in the anti-tumor effects of curcumin by activating the adenosine monophosphate-activated protein kinase and inactivating mTOR signaling [22].

In the present study, we suspected that the lncRNA NBR2 functions as a tumor suppressor in CRC development. Thus, we aimed to investigate the regulatory effects of the lncRNA NBR2 on macrophage polarization and the consequent anti-tumor effects against CRC. By clarifying the function of the lncRNA NBR2 in macrophage polarization, a novel biomarker and drug target can be determined for the diagnosis and treatment of clinical CRC.

## Materials and methods

**Tissues and cell lines**. Ten pairs of tumor and para-carcinoma tissues from 10 CRC patients and HCT116, SW480, and THP-1 cells were purchased from the American Type Culture Collection (ATCC, Rockville, MD, USA). Dulbecco’s Modified Eagle Medium (DMEM) with 10% fetal bovine serum was used to culture the cells at 37°C with 5% CO_2_.

**Reverse transcription polymerase chain reaction (qRT-PCR)**. Total RNA was collected from the tissues or cells using an RNA Extraction Kit (Thermo Fisher Scientific, Waltham, USA) according to the manufacturer’s instructions. The extracted RNA was quantified using a NanoDrop spectrophotometer (Thermo Fisher Scientific). A specific RT primer was used to reverse transcribe the complementary DNA. SYBR Premix Ex TaqTM (Thermo Fisher Scientific) with an Applied Bio-Rad CFX96 Sequence Detection system (GenScript Biotechnology Co. Ltd., Nanjing, China) was used for real-time PCR. The expression level of NBR2 was determined using the threshold cycle (Ct), and the relative expression levels were calculated using the 2^−ΔΔCt^ method after normalization with GAPDH [23]. Three independent assays were performed. The information on the primers is shown in Table 1.

**Table 1.** The sequences of primers for NBR2 and GAPDH

Primer ID	Sequences (5ʹ-3ʹ)
NBR2 F	GGAGGTCTCCAGTTTCGGTA
NBR2 R	TTGATGTGTGCTTCCTGGG
GAPDH F	CAATGACCCCTTCATTGACC
GAPDH R	GAGAAGCTTCCCGTTCTCAG

**Western blot assay**. Total proteins were isolated from tissues or cells using the Nuclear and Cytoplasmic Protein Extraction Kit (Thermo Fisher Scientific). Approximately 40 μg of protein was separated on a 12% SDS-polyacrylamide gel, and the gel was transferred to a polyvinylidene difluoride membrane (Millipore, Massachusetts, USA). The membrane was blocked with 5% nonfat dry milk in Tris-buffered saline with 0.1% Tween-20 (pH 7.4) for 1 h at room temperature and incubated overnight with primary rabbit anti-human antibodies against TNF-α (1:1000), HLA-DR (1:1000), Arg-1 (1:1000), CD163 (1:1000), CD206 (1:1000), and GAPDH (1:1000) (Abcam, Cambridge, MA, USA). A horseradish peroxidase-conjugated antibody against rabbit IgG (1:5000, Abcam) was used as the secondary antibody. Blots were incubated with ECL reagents (Amersham Pharmacia Biotech, Inc., USA) and exposed to Tanon 5200-multi to detect protein expression. Three independent assays were performed [24].

**Transfection**. THP-1 cells were incubated with 350 nM phorbol 12-myristate 13-acetate (PMA) for 24 h to induce the differentiation of M0 macrophages. A lentivirus particle containing pcDNA-NBR2 or siRNA-NBR2 (siR-NBR2) was designed and prepared by GenScript Biotechnology Co. Ltd. 24 h prior to transfection. M0 macrophages were seeded in a 6-well culture plate. Lentivirus particles were transfected using Lipofectamine 2000 (Invitrogen, Carlsbad, CA, USA) and X-treme GENE HP DNA Transfection Reagent (Invitrogen) according to the manufacturer’s instructions. After 24 h or 48 h, the cells were collected and subjected to further analysis. The assays were performed in triplicate, and more than nine wells were treated with the same type of lentivirus particles. The lentivirus particle containing the negative control (NC) sequence was used as a control.

**Transwell Migration Assay** [25]. Approximately 5 × 10^5^ HCT116 or SW480 cells were suspended in serum-free DMEM and plated into the upper insert of a six-well Transwell plate, and another 5 × 10^5^ cells were suspended in the lower chamber. The cells were incubated at 37°C for 8 h. The non-migratory cells in the upper layer were removed, and the migratory cells were fixed with 4% paraformaldehyde at room temperature for 10 min, followed by staining with crystal violet solution. Images were photographed under a light microscope (Olympus, Tokyo, Japan) and quantified by counting the number of cells in five randomly selected fields of view for each well.

**CCK8 cell proliferation assay**. CCK8 assay (CCK8, Sigma) was used to test the proliferation of HCT116 and SW480 cells according to the manufacturer’s instructions. Briefly, 10^4^–10^5^ cells/well in 100 μL of culture medium were seeded in a 96-well plate and incubated for 24 h at 37°C in a humidified incubator with 5% CO_2_. Then, 10 μL of various concentrations of drugs were added to the plate, and the treated cells were incubated for an appropriate length of time (e.g., 24 h), and 10 μL of CCK-8 solution was added into each well using a repeating pipette. The plate was incubated for 1–4 h, and the absorbance at 450 nm was measured using a benchmark microplate reader (Bio-Rad, CA, USA) [19]. Three independent assays were performed.

**Enzyme-linked immunosorbent assay**. According to the manufacturer’s instructions (Sigma), the concentrations of IL-1β, IL-6, and TNF-α in the cells or tumor tissues were determined by enzyme-linked immunosorbent assay (ELISA). The procedure includes sample addition, enzyme addition, incubation, working solution preparation, washing, dyeing, termination, and detection. The linear regression equation was described based on the concentration of standards and the OD value. The concentrations of the samples were calculated according to the equation, detected OD value, and dilution factor [26].

**Xenograft experiments**. Twelve female BALB/c nude mice were purchased from Beijing Vital River Laboratory Animal Technology Co., Ltd. The animals were randomly divided into four groups. The animals were injected subcutaneously with HCT116 cells (or SW480 cells), HCT116 cells (or SW480 cells) co-cultured with M0 macrophages, HCT116 cells (or SW480 cells) co-cultured with M0 macrophages transfected with pcDNA-NC, or HCT116 cells (or SW480 cells) co-cultured with M0 macrophages transfected with pcDNA-NBR2. The concentration of planted cells was 3 × 10^6^ cells in 0.2 mL phosphate buffered saline. The animals were monitored for 18 days post-injection. The length (L) and width (W) of the tumor were measured and recorded every 2 days after the cells were injected. The volume of the tumor (V) was calculated using the following formula: V = L × W2 × 0.5. After 18 days of treatment, the animals were sacrificed with CO_2_, and the tumors were collected and weighed [27].

**Statistical analysis**. Statistically significant differences for continuous variables were determined using a one-way analysis of variance (ANOVA) with least significant difference (LSD) test for normally distributed data. All tests were performed using the GraphPad Prism 5 software. Statistical significance was set at P < 0.05.

**Ethics Statements**. All animal experiments involved in this manuscript were authorized by the ethical committee of the University of Chinese Academy of Sciences and performed according to the guidelines for care and use of laboratory animals, as well as to the principles of laboratory animal care and protection.

## Results

We suspected that the lncRNA NBR2 functions as a tumor suppressor in the development of CRC. The present study aimed to investigate the regulatory effects of the lncRNA NBR2 on macrophage polarization and the consequent anti-tumor effects against CRC. The function of the lncRNA NBR2 in regulating M1 polarization and the anti-tumor efficacy of the lncRNA NBR2 was investigated and confirmed by establishing the lncRNA NBR2 overexpressed and knocked down CRC cells. Then, we explored (1) whether the lncRNA NBR2 is a tumor suppressor, (2) the regulatory effects of the lncRNA NBR2 on the polarization of TAM, and (3) the inhibitory effects of the lncRNA NBR2 on the growth of CRC cells both *in vitro* and *in vivo*.

**Figure 1.**
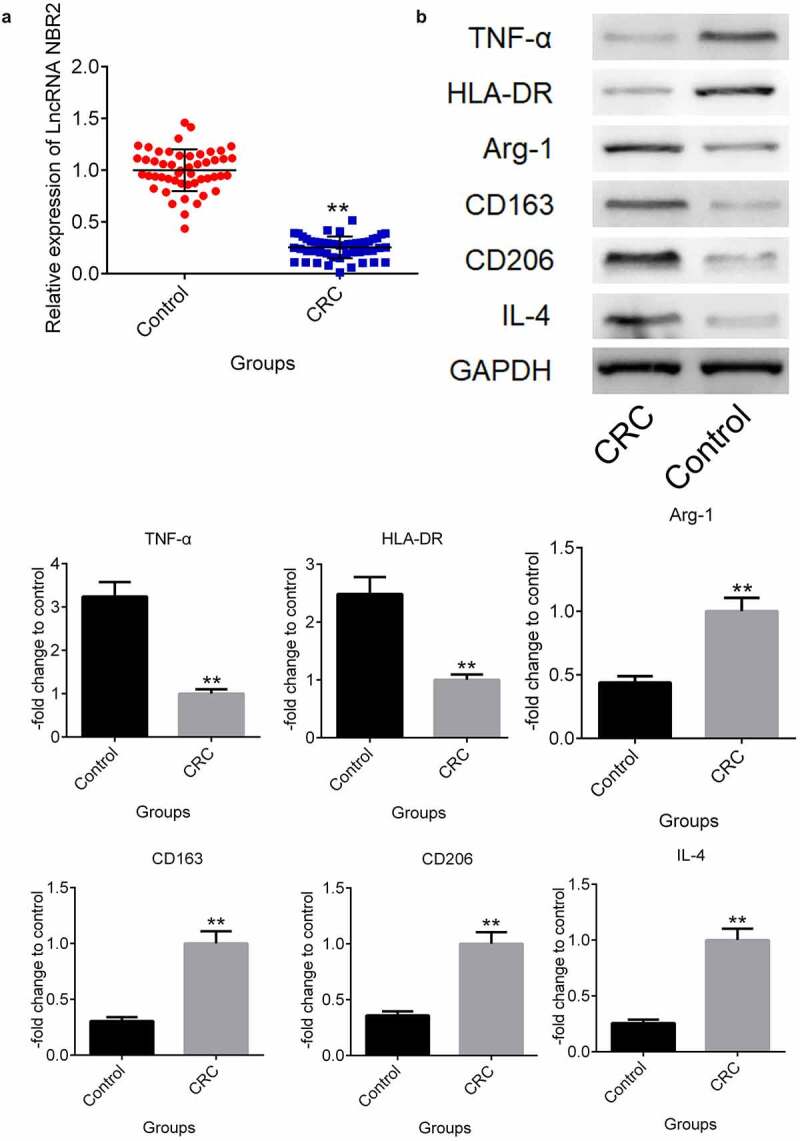
The lncRNA NBR2 was downregulated and M2 polarization was abundant in CRC patients. A) The expression of the lncRNA NBR2 in the tissues was evaluated by qRT-PCR. B) The expression level of TNF-α, HLA-DR, Arg-1, CD163, CD206, and IL-4 in the tissues was determined by western blot (**P < 0.01, vs. Control)

**In CRC patients, the lncRNA NBR2 was downregulated, and M2 polarization was abundant**. Ten pairs of tumor and para-carcinoma tissues from 10 CRC patients were collected to check the difference in the lncRNA NBR2 expression levels. The para-carcinoma tissues were used as the control group. As shown in Figure 1a, the lncRNA NBR2 was found to be significantly downregulated in the isolated tumor tissues from CRC patients compared to the para-carcinoma tissues (**P < 0.01, vs. Control). To compare the M1 and M2 polarization in the tumor and para-carcinoma tissues, the expression levels of M1 and M2 macrophage markers and the polarization inducer were evaluated in clinical tumor and para-carcinoma tissues. As shown in Figure 1b, the M1 microphage markers TNF-α and HLA-DR were greatly downregulated in tumor tissues, compared with para-carcinoma tissues. Significantly higher expression levels of the M2 microphage markers Arg-1, CD163, and CD206 were observed in tumor tissues than in para-carcinoma tissues, as well as the upregulated IL-4 in tumor tissues, which is an inducer of polarization from M2 macrophages to M1 macrophages (**P < 0.01, vs. Control).

**Figure 2.**
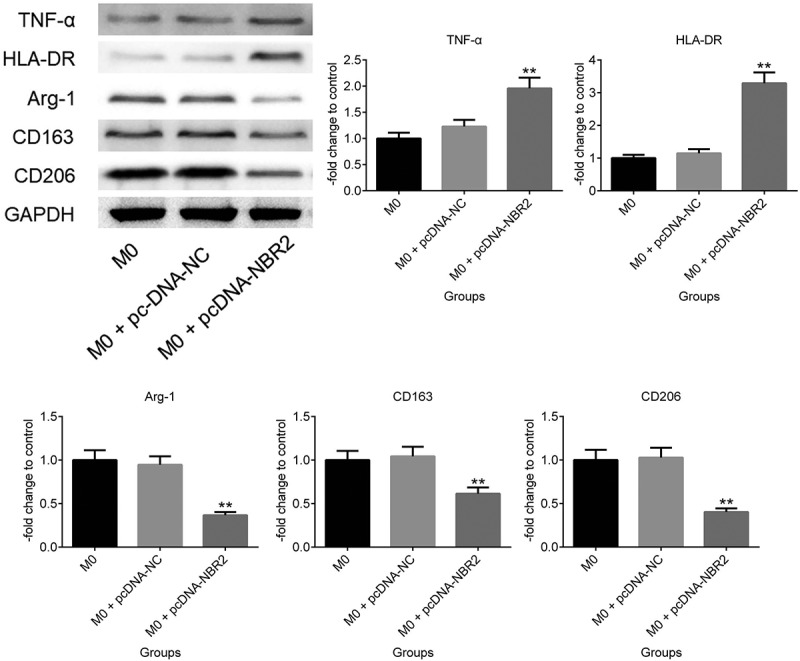
The expression level of TNF-α, HLA-DR, Arg-1, CD163, and CD206 in macrophages was determined by western blot (**P < 0.01, vs. M0)

**M1 polarization was induced by upregulating the lncRNA NBR2**. To investigate the regulatory function of the lncRNA NBR2 on macrophage polarization, M0 macrophages were induced by incubating the THP-1 cells with 350 nM PMA and transfecting pcDNA-NBR2 into the M0 macrophages to promote the expression level of the lncRNA NBR2 in the macrophages. As shown in Figure 2, TNF-α and HLA-DR were found to be significantly upregulated in pcDNA-NBR2 transfected macrophages, compared with M0 macrophages. However, the expression levels of Arg-1, CD163, and CD206 were significantly suppressed in pcDNA-NBR2 transfected macrophages, compared with M0 macrophages (**P < 0.01, vs. M0). These data indicated that M1 polarization was activated and M2 polarization was inhibited by upregulating the expression level of the lncRNA NBR2 in M0 macrophages.

**The lncRNA NBR2 inhibited the proliferation and metastasis of HCT116 and SW480 cells by enhancing the function of macrophages**. To fully investigate the function of the lncRNA NBR2 in the development and progression of CRC cells, the lncRNA NBR2 overexpressed macrophages and the lncRNA NBR2 knockdown macrophages were established by transfecting macrophages with pcDNA3.1-NBR2 and siRNA against the lncRNA NBR2, respectively.

**Figure 3.**
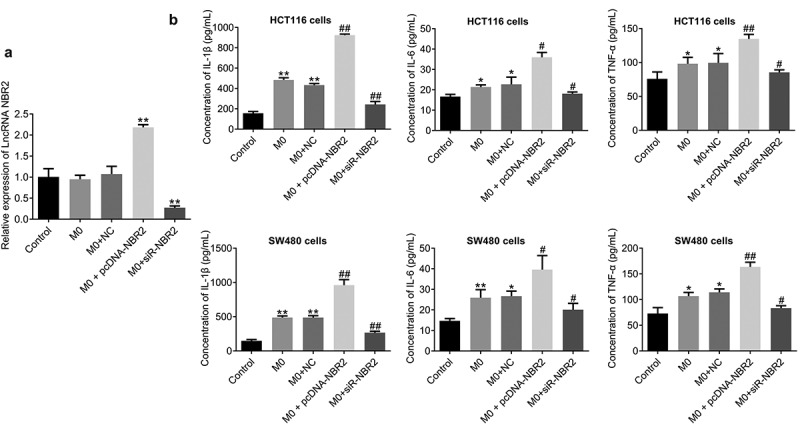
The lncRNA NBR2 induced the production of inflammatory factors. A) The expression of the lncRNA NBR2 was evaluated by qRT-PCR (**P < 0.01, vs. Control). B) The production of IL-1β, IL-6, and TNF-α in the supernatant of treated HCT116 and SW480 cells was determined by ELISA (*P < 0.05, vs. Control; **P < 0.01, vs. Control; ^#^P < 0.01, vs. M0; ^##^P < 0.01, vs. M0)

First, as shown in Figure 3a, the lncRNA NBR2 was highly expressed in macrophages transfected with pcDNA-NBR2 and downregulated in those transfected with siR-NBR2, which was confirmed by the results of qRT-PCR (**P < 0.01, vs. Control). By determining the concentration of inflammatory factors in the supernatant, we found that the concentration of IL-1β, IL-6, and TNF-α (Figure 3b) was greatly increased in HCT116 cells or SW480 cells incubated with M0 macrophages and M0 macrophages transfected with NC (**P < 0.01, vs. Control), which was further increased in HCT116 or SW480 cells incubated with M0 macrophages transfected with pcDNA-NBR2 and dramatically declined in HCT116 cells or SW480 cells incubated with M0 macrophages transfected with siR-NBR2 (^##^P < 0.01, vs. M0).

**Figure 4.**
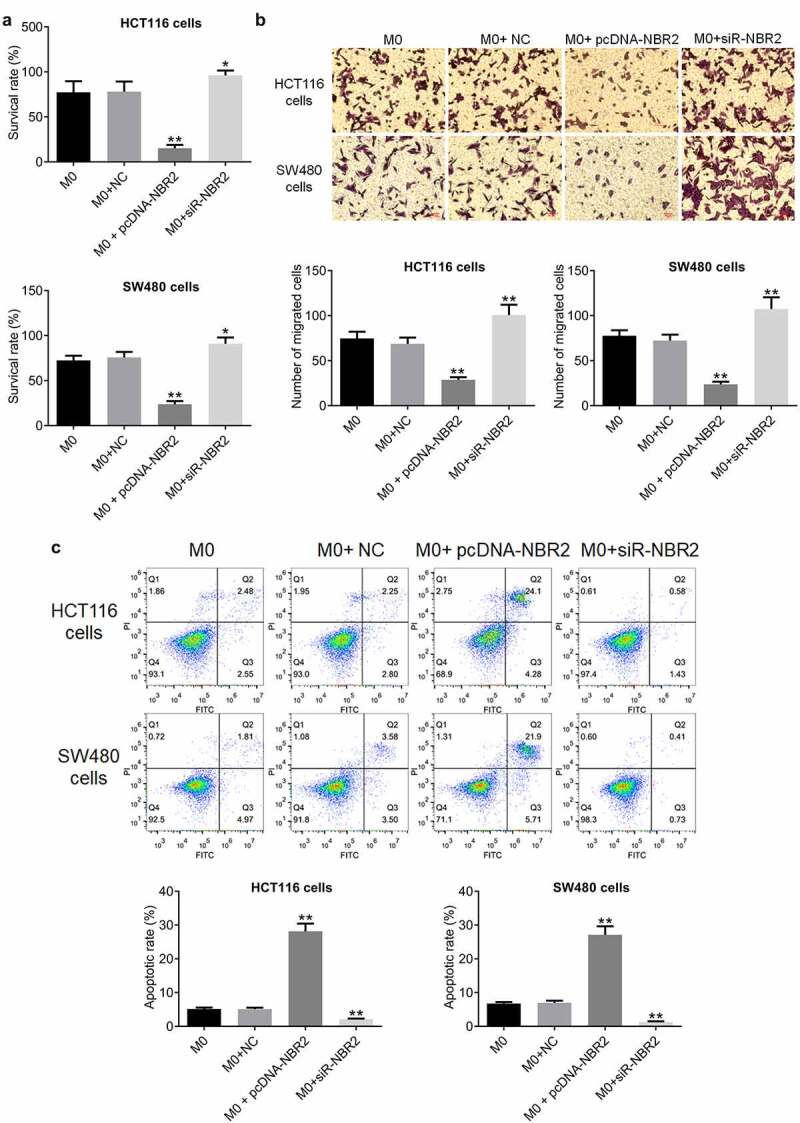
The lncRNA NBR2 inhibited the proliferation and metastasis of HCT116 and SW480 cells. A) The proliferation of HCT116 and SW480 cells was detected by CCK8 assay (*P < 0.05, vs. M0; **P < 0.01, vs. M0; respectively). B) The migration ability of HCT116 and SW480 cells was evaluated by Transwell assay (**P < 0.01, vs. M0). C) The apoptosis of HCT116 and SW480 cells was determined by the flow cytometry assay (**P < 0.01, vs. M0)

To investigate the effects of macrophages transfected with pcDNA-NBR2 or siR-NBR2 on the proliferation of CRC cells, HCT116 or SW480 cells were incubated with M0 macrophages, M0 macrophages transfected with NC, M0 macrophages transfected with pcDNA-NBR2, or M0 macrophages transfected with siR-NBR2. As shown in Figure 4a, the survival rate of HCT116 cells or SW480 cells detected by CCK8 assay was decreased greatly by the co-incubation of M0 macrophages transfected with pcDNA-NBR2 and elevated significantly by the co-incubation of M0 macrophages transfected with si-NBR2, compared to M0 macrophages (**P < 0.01, vs. M0).

The results of migration study are shown in Figure 4b. The migration rate of HCT116 and SW480 cells was decreased greatly by co-incubating with M0 macrophages transfected with pcDNA-NBR2 and promoted by the co-incubation of M0 macrophages transfected with si-NBR2, compared with M0 macrophages (*P < 0.05, vs. M0; **P < 0.01, vs. M0), indicating an inhibitory effect on the metastasis of CRC cells by NBR2-overexpressed macrophages.

The apoptotic rate of CRC cells was determined by flow cytometry. As shown in Figure 4c, in HCT116 cells, the apoptotic rate was 5.03% and 5.05% after co-incubation with M0 macrophages and M0 macrophages transfected with NC, respectively, which was significantly elevated to 28.38% after the co-incubation with M0 macrophages transfected with pcDNA-NBR2 and declined to 2.01% after the co-administration of M0 macrophages transfected with si-NBR2, respectively (**P < 0.01, vs. M0). In addition, in SW480 cells, the apoptotic rate was 6.78% and 7.08% after co-incubation of M0 macrophages and M0 macrophages transfected with NC, respectively, which was significantly elevated to 27.61% after the co-incubation with M0 macrophages transfected with pcDNA-NBR2 and declined to 1.14% after the co-administration of M0 macrophages transfected with si-NBR2, respectively (**P < 0.01, vs. M0).

**Figure 5.**
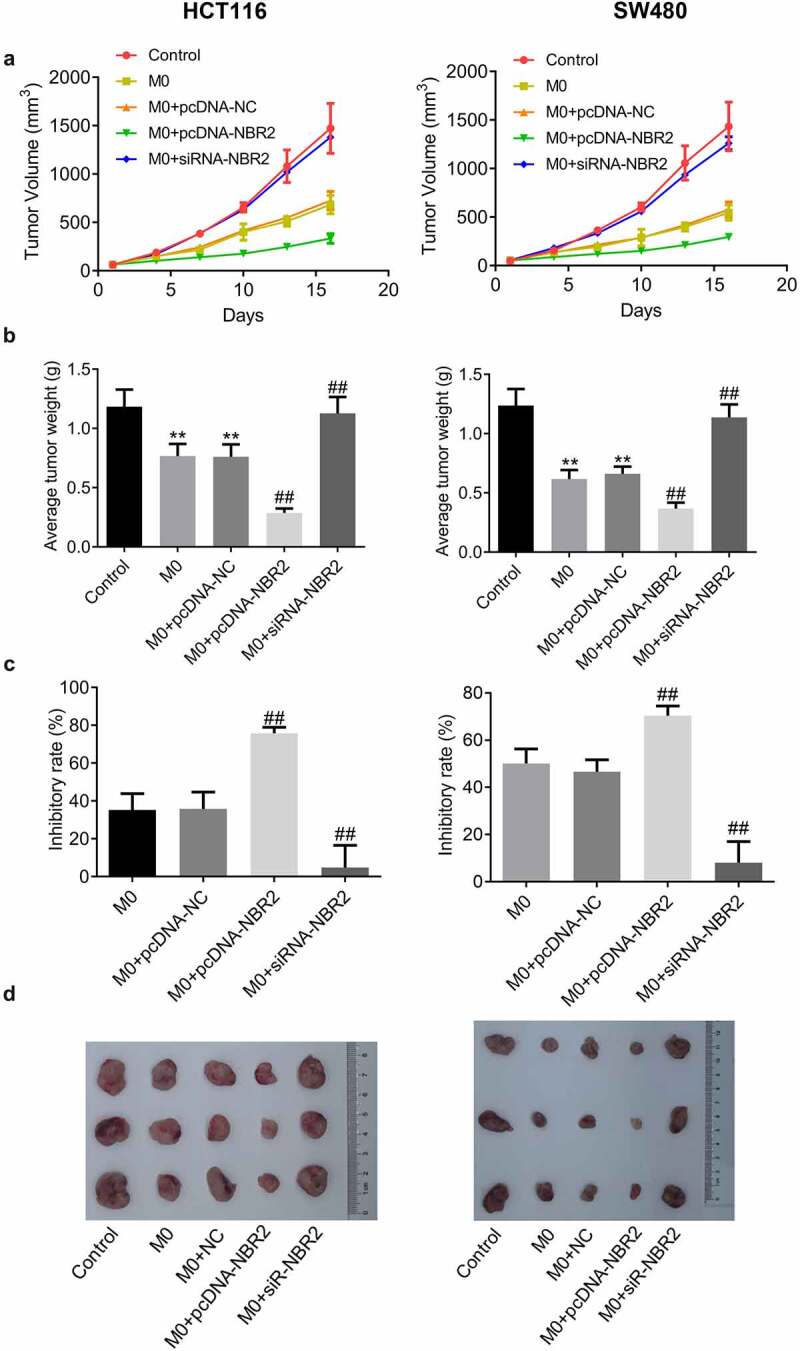
The lncRNA NBR2 suppressed the tumor growth in the HCT116 and SW480 xenograft model. A) Curves of tumor volume versus injection time; B) Average tumor weight in mice (**P < 0.01, vs. Control; ^##^P < 0.01, vs. M0). C) The inhibitory rate of tumor growth (^##^P < 0.01, vs. M0). D) Photos of tumors at the end of the study

**The lncRNA NBR2 suppressed the tumor growth of HCT116 and SW480 cells in the xenograft model**. To evaluate the effects of NBR2-overexpressed macrophages and NBR2-knockdown on *in vivo* growth of CRC tumor, HCT116 cells (or SW480 cells), HCT116 cells (or SW480 cells) co-incubated with M0 macrophages, HCT116 cells (or SW480 cells) co-incubated with M0 macrophages transfected with NC, HCT116 cells (or SW480 cells) co-incubated with M0 macrophages transfected with pcDNA-NBR2, and HCT116 cells (or SW480 cells) co-incubated with M0 macrophages transfected with siR-NBR2 were injected subcutaneously into the nude mice to establish the xenograft models. We monitored the dynamic change in tumor volume for 18 days after planting (Figure 5a). In both the HCT116 and SW480 xenograft models, the smallest tumor volume was observed in the M0+ pcDNA-NBR2 group, and the largest tumor volume was observed in the M0+ siR-NBR2 group, respectively. As shown in Figure 5b, in both the HCT116 and SW480 xenograft models, the average tumor weight isolated from M0 and M0+ NC group was much heavier than that from the control group (**P < 0.01, vs. Control), which was significantly lower in the M0+ pcDNA-NBR2 group and further elevated in the M0+ siR-NBR2 group, respectively (^##^P < 0.01 M0). In the HCT116 xenograft model, the inhibitory rate (Figure 5c) in the M0, M0+ NC, M0+ pcDNA-NBR2, and M0+ siR-NBR2 groups were 35.2%, 35.7%, 75.8% and 4.79%, respectively (^##^P < 0.01, vs. M0). In the SW480 xenograft model, the inhibitory rates in the M0, M0+ NC, M0+ pcDNA-NBR2, and M0+ siR-NBR2 groups were 50.1%, 46.6%, 70.4% and 8.1%, respectively (^##^P < 0.01, vs. M0). Representative images of the tumors are shown in Figure 5d.

**Figure 6.**
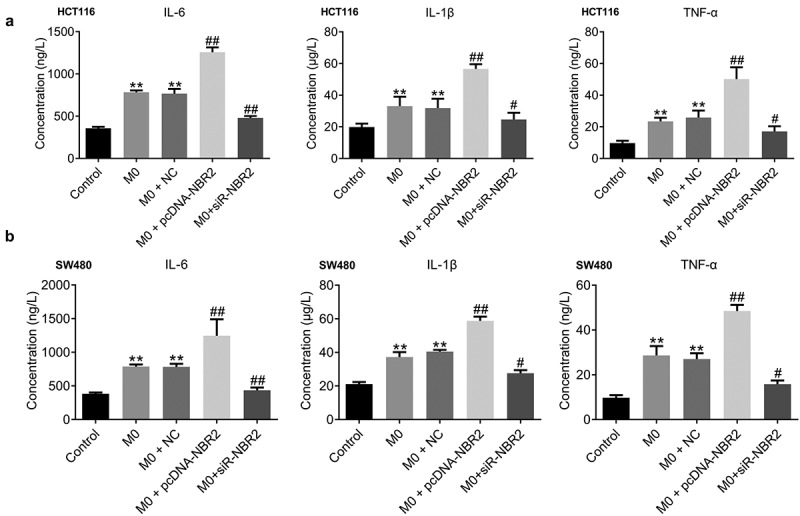
The inflammation in tumor tissues was activated by the lncRNA NBR2. A) The production of IL-1β, IL-6, and TNF-α in HCT116 tumor tissues was measured by the ELISA (**P < 0.01, vs. Control: ^#^P < 0.05, vs. M0; ^##^P < 0.01, vs. M0). B) The production of IL-1β, IL-6, and TNF-α in SW480 tumor tissues was measured by the ELISA (**P < 0.01, vs. Control; ^#^P < 0.05, vs. M0; ^##^P < 0.01, vs. M0)

**M1 polarization was activated in the M0+ pcDNA-NBR2 group and inactivated in the M0+ siR-NBR2 group**. Firstly, we determined the concentration of inflammatory factors in the tumors from each group by ELISA. As shown in Figure 6, in both the HCT116 and SW480 xenograft models, IL-1β, IL-6, and TNF-α were highly produced in the M0 and M0+ NC groups, compared to the control (**P < 0.01, vs. Control), which was greatly promoted in the M0+ pcDNA-NBR2 group and significantly declined in the M0+ siR-NBR2 group (^#^P < 0.05, M0; ^##^P < 0.01, M0).

**Figure 7.**
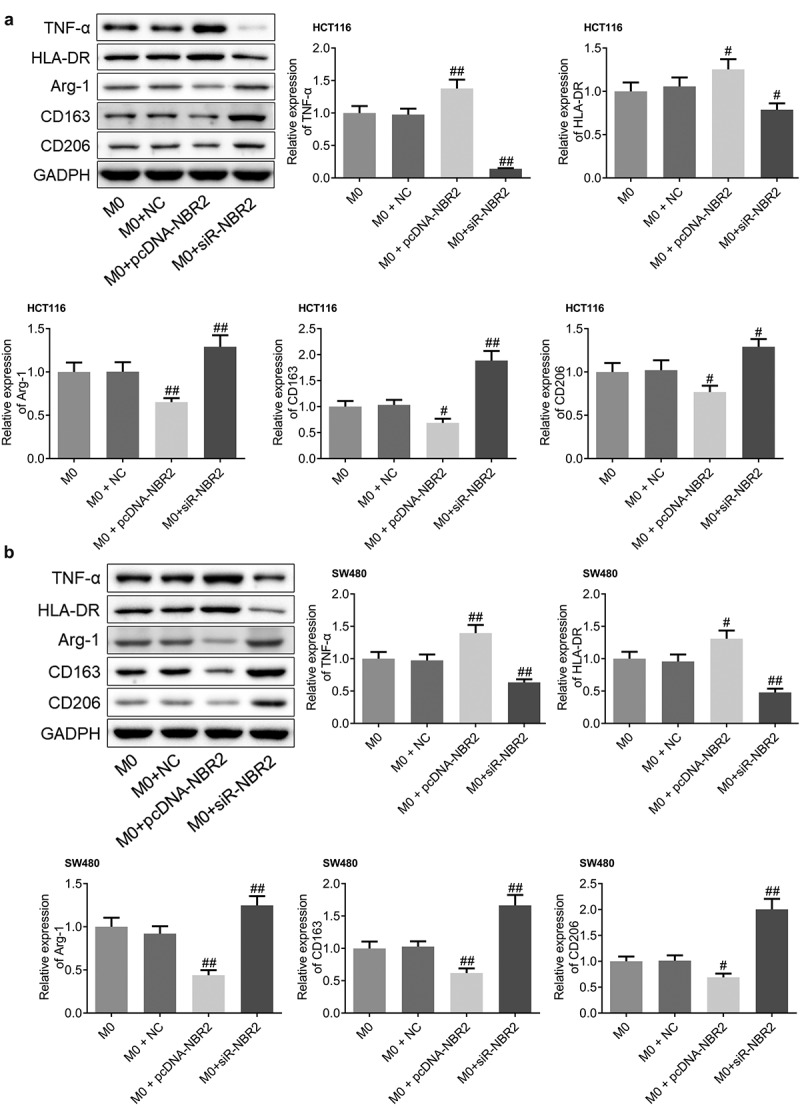
M1 polarization in CRC tissues was activated by the lncRNA NBR2. A) The expression level of TNF-α, HLA-DR, Arg-1, CD163, and CD206 in HCT116 tumor tissues was determined by western blot (^#^P < 0.05, vs. M0; ^##^P < 0.01, vs. M0). B) The expression level of TNF-α, HLA-DR, Arg-1, CD163, and CD206 in SW480 tumor tissues was determined by western blot (^#^P < 0.05, vs. M0, ^##^P < 0.01, vs. M0)

To investigate the polarization state of macrophages in the tumor tissues from each group, the expression levels of TNF-α, HLA-DR, Arg-1, CD163, and CD206 were determined in the tumor tissues. As shown in Figure 7, we found that in both the HCT116 and SW480 xenograft models, compared to M0, TNF-α and HLA-DR were significantly upregulated and Arg-1, CD163, and CD206 were greatly downregulated in the M0+ pcDNA-NBR2 group, while TNF-α and HLA-DR were dramatically downregulated and Arg-1, CD163, and CD206 were greatly upregulated in the M0+ siR-NBR2 group (^#^P < 0.05, vs. M0; ^##^P < 0.01, vs. M0). These data suggests the activation of M1 polarization in the M0+ pcDNA-NBR2 group and the inactivation of M1 polarization in the M0+ siR-NBR2 group.

## Discussion

More evidences have been reported claiming that the interaction between tumor cells and tumor microenvironment is essential for the proliferation and metastasis of malignant tumor [28]. The local microenvironment is changed and maintained by tumor cells for better survival and development through autocrine and paracrine mechanisms. Correspondingly, the proliferation and metastasis of tumor cells can be induced by the tumor microenvironment by changing the functions of metabolism, secretion, and immunity [29]. The tumor microenvironment mainly consists of extracellular matrix, soluble molecules, and tumor stromal cells. Tumor cells tend to change the surrounding environment by inducing phenotypic modulation of tumor stromal cells to form a dynamic internal environment led by tumor stromal cells [30,31]. Among these tumor stromal cells, the activation of immune and inflammatory cells is very important for the establishment of the tumor microenvironment. TAMs have been proven to be an important member in the family of tumor stromal cells, which accounts for approximately 50% of the total tumor stromal cells. TAM is derived from monocytes or macrophages from normal tissues and plays an important role in the process, development, metastasis, and immune escape of malignant tumors by secreting multiple types of inflammatory factors [32,33]. Therefore, TAM may be the next breakthrough in the treatment of malignant tumors.

The macrophage balance hypothesis, proposed by Mantocani in 1992, indicates that both tumor-killing and -inducing activities are involved in TAM [34]. Activated macrophages can be classified into two categories based on differences in molecular phenotype and biological function: M1 and M2 macrophages. The differentiation of TAM to M1 or M2 macrophages is called polarization [35]. The opposite effects on the tumor microenvironment have been reported between M1 and M2 macrophages. M1 macrophages, also known as classically activated macrophages, are produced from the differentiation of monocytes induced by IFN-γ and LPS. Pathogenic microorganisms and tumor cells can be eliminated by M1 macrophages by acute proinflammatory response, immune activation reaction, and cytophagy through the release of proinflammatory factors, such as NO, IL-1β, IL-6, TNF-α, and chemokines such as CCL2, CCL3, CCL5, CXCL9, and CXCL10 [35,36].

In the present study, the relative proportion of M1 macrophages in CRC patients was evaluated by determining the expression levels of TNF-α and HLA-DR [37]. We found that less M1 polarization was involved in CRC tissues than in para-carcinoma tissues, indicating that the differentiation of M0 macrophages to M1 macrophages was inhibited by the CRC cells. When the proportion of M1 macrophages was promoted by upregulating the expression of the lncRNA NBR2, increased production of inflammatory factors and decreased tumor cell proliferation and metastasis were observed in the co-culture system of HCT116 cells and macrophages or SW480 cells and macrophages. Elevated tumor cell proliferation and metastasis were also observed in the co-culture system of HCT116 and SW480 cells and NBR2-knockdown macrophages. Furthermore, a significant inhibitory effect on tumor growth in the nude mice xenograft model was observed in the lncRNA NBR2 overexpressed group, as well as the promotion of M1 macrophage proportion in the tumor tissues. The tumor growth of CRC cells was significantly promoted in the lncRNA NBR2 knockdown group, accompanied by a decline in the proportion of M1 macrophages in the tumor tissues. These data indicated that M1 polarization was significantly induced by the lncRNA NBR2, which exerted significant anti-tumor effects both *in vitro* and *in vivo*.

M2 macrophages, also known as alternatively activated macrophages, are mainly differentiated from monocytes through the induction of IL-4, IL-13, PGE2, or TGF-β. The proliferation and metastasis of tumor cells can be promoted by M2 macrophages by releasing anti-inflammatory factors such as IL-10 and TGF-β [38], immunosuppressive factors such as PGE2, arginase-I [39], somatomedins such as EGF, CCL18, and HIF-1α [40] and pro-metastasis factors such as MMPs, uPA, and uPAR [41]. In the present study, the relative proportion of M2 macrophages in CRC patients was determined by detecting the expression levels of its biomarkers, Arg-1, CD163, and CD206 [42].

In the present study, a higher M2 polarization was observed in CRC tissues than in para-carcinoma tissues, indicating that the polarization of M0 macrophages to M2 macrophages was promoted by CRC cells. By upregulating the expression of the lncRNA NBR2, we found that the proportion of M2 macrophages was greatly suppressed. Increased production of inflammatory factors and decreased tumor cell proliferation and metastasis were observed in the co-culture system of HCT116 cells, SW480 cells, and macrophages. Elevated tumor cell proliferation and metastasis were also observed in the co-culture system of HCT116 and SW480 cells and NBR2-knockdown macrophages. In addition, a significant inhibitory effect on tumor growth in the nude mice xenograft model was observed in the lncRNA NBR2 over-expressing group, as well as a decrease in the proportion of M2 macrophages in the tumor tissues. The tumor growth of CRC cells in the nude mice xenograft model was significantly promoted in the lncRNA NBR2 knockdown group, accompanied by an increase in the proportion of M2 macrophages in the tumor tissues. These data indicated that M2 polarization could be suppressed by upregulating the lncRNA NBR2 in the macrophages, which prevented the proliferation and metastasis of CRC cells. However, further investigation on the mechanism underlying the effects of the lncRNA NBR2 on the polarization of macrophages is necessary. Regulatory signaling pathways may be involved in the polarization, and the possible correlation between the lncRNA NBR2 and the signaling pathways should be explored.

## Conclusion

Our findings indicated that the lncRNA NBR2 might suppress the progression of colorectal cancer *in vitro* and *in vivo* by regulating the polarization of TAM, which could be developed as an important biomarker for the diagnosis and treatment of clinical CRC.

## Highlights

The lncRNA NBR2 is a tumor suppressor.

The lncRNA NBR2 facilitates the M1 polarization of macrophages.

The lncRNA NBR2 inhibits the growth of colorectal cancer.
